# Identification of HIPK3 as a potential biomarker and an inhibitor of clear cell renal cell carcinoma

**DOI:** 10.18632/aging.202294

**Published:** 2021-01-20

**Authors:** Wen Xiao, Tao Wang, Yuzhong Ye, Xuegang Wang, Bin Chen, Jinchun Xing, Hongmei Yang, Xiaoping Zhang

**Affiliations:** 1 Department of Urology, Union Hospital, Tongji Medical College, Huazhong University of Science and Technology, Wuhan, China; 2 Department of Urology, The First Affiliated Hospital, School of Medicine, Xiamen University, Xiamen, Fujian, China; 3 Department of Pathogenic Biology, School of Basic Medicine, Huazhong University of Science and Technology, Wuhan, China

**Keywords:** ccRCC, biomarker, HIPK3, prognostic indicator

## Abstract

Invasion and metastasis are the main causes of poor prognosis in patients with clear cell renal cell carcinoma (ccRCC). The homeodomain interacting protein kinases (HIPKs) can regulate cell proliferation and apoptosis. Little is known about the prognostic role of HIPKs in ccRCC. Here we use Kaplan-Meier survival analysis and multivariate analysis to analyze the correlation of overall survival (OS) and disease–free survival (DFS). ROC curves analyzed the relationship between clinicopathological parameters and HIPK3 expression in ccRCC. Univariate analysis and multivariate analysis confirmed that the expression of HIPK3 was associated with OS (HR, 0.701; P=0.041) and DFS (HR, 0.630; P=0.012). Low HIPK3 expression was a poor prognostic factor and HIPK3 expression was significantly down-regulated in ccRCC cancer tissues when compared with normal renal tissues. *In vitro* cell results also confirmed that HIPK3 over-expression could inhibit tumor growth and malignant characteristics. The results indicate that low expression of HIPK3 in ccRCC tissues is significantly associated with poor survival rates in tumor patients, and HIPK3 may be used as a valuable biomarker and inhibitor of ccRCC.

## INTRODUCTION

Kidney cancer or renal cell cancer (RCC) was one of the top ten leading cancer types for new cancer cases (estimated about 73,750) and deaths (estimated about 14,830) in 2020 in the United States [1]. Global statistics found approximately 403,262 (2.2%) new cases of RCC and 175,098 (1.8%) deaths in 2018 [2]. Clear cell renal cell carcinoma (ccRCC) is still a deadly disease and closely related to cancer-related deaths in the urinary system [3]. Early and timely diagnosis of tumors is an extremely important part of clinical treatment management. Therefore, it is one of the hot spots of current research.to explore new potential molecules and probable mechanisms for the occurrence and development of ccRCC 90% of ccRCC cancer deaths are related to cancer metastasis and invasion [4, 5]. Nephrectomy is currently the main method for early treatment of ccRCC, but there is still a high risk of recurrence after nephrectomy [6]. Targeted therapy with tyrosine kinase inhibitor (TKI) drugs has shown very encouraging therapeutic effects in patients with metastatic or advanced ccRCC [7, 8]. Unfortunately, the existence of TKI resistance can still lead to further tumor development [9]. The phosphorylation of extracellular signal regulated kinase (p-ERK) pathway was associated with poor prognosis of ccRCC patients [10, 11]. Persistent activation or reactivation of ERK signaling mediates TKI resistance in various tumors [12–16]. Therefore, it is urgent to explore more effective prognostic molecular biomarkers and identify new drug targets for new ccRCC treatments.

In the current study, we investigated the relationship between clinicopathological features and patient survival rates and the expression of 4 HIPK members in the TCGA database. The results show that low expression of HIPK3 could be a predictor of poor overall survival (OS) and disease-free survival (DFS) with univariate and multivariate analysis in ccRCC, and can be used as a tumor suppressor gene among 4 HIPKs. The expression and function of HIPK3 may be associate with BAP1 mutation or expression of TWIST1, CDH1(E-Cadherin), Vimentin. We then confirmed that HIPK3 expression in ccRCC tissues was lower than that in normal kidney tissues, and overexpression of HIPK3 could inhibit the proliferation, migration, and invasion of renal cancer cells. The analysis and verification results showed that HIPK3 can be used as a novel prognostic marker of ccRCC and may be a therapeutic target with certain potential clinical application value.

## RESULTS

### Relative expression of HIPK family in ccRCC

**Figure 1:**
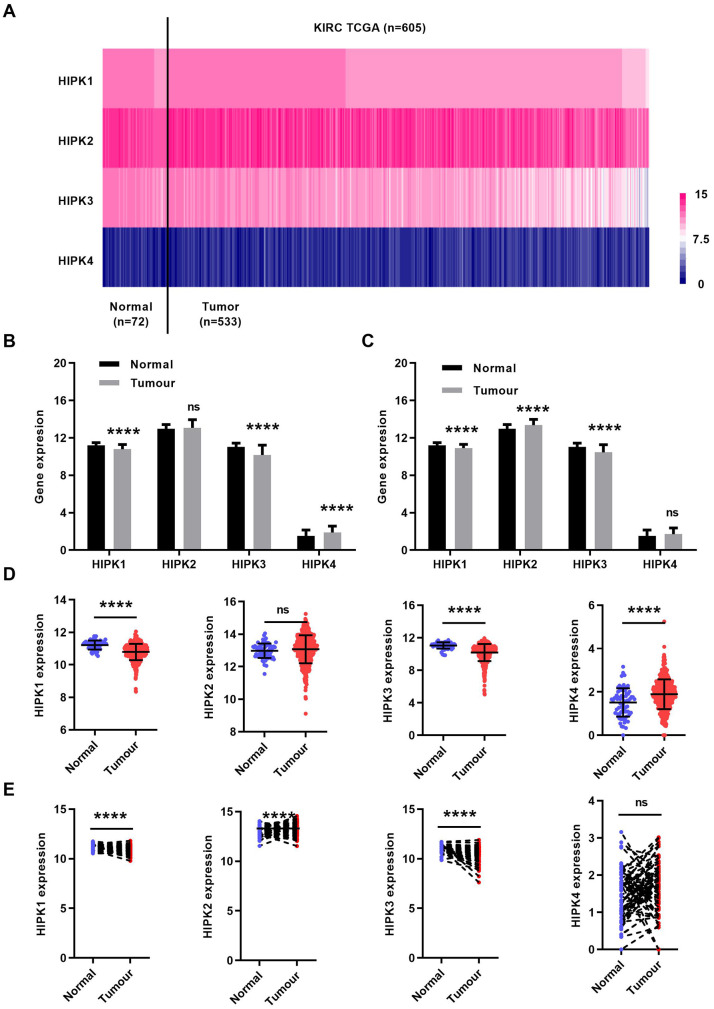
**HIPK family expression in TCGA-KIRC microarray datasets.** (**A**) Heat map depicting HIPKs expression in TCGA-KIRC microarray datasets (n=605). (**B**) Relative HIPKs expression in TCGA-KIRC. Red indicates high expression; white indicates medium expression; blue indicates low expression. HIPK, Homeodomain interacting protein kinases; TCGA-KIRC, The Cancer Genome Atlas kidney renal clear cell carcinoma. ****P<0.0001.

First, we analyzed the mRNA expression level of 4 HIPK family members in the TCGA-KIRC database. The heat map showed the expression levels of HIPK family members in Figure 1A. The expression of HIPK family members in total ccRCC cancer tissues (N=533) and non-cancerous normal tissues (N=72) was shown in Figure 1B, 1D. Expression of paired ccRCC tissues and non-cancerous normal tissues (N=72) was shown in Figure 1C, 1E. HIPK1 and HIPK3 had a lower expression in tumor tissues than that in corresponding non-cancerous normal tissues.

### Prognostic significance of HIPK family members in ccRCC

**Figure 2:**
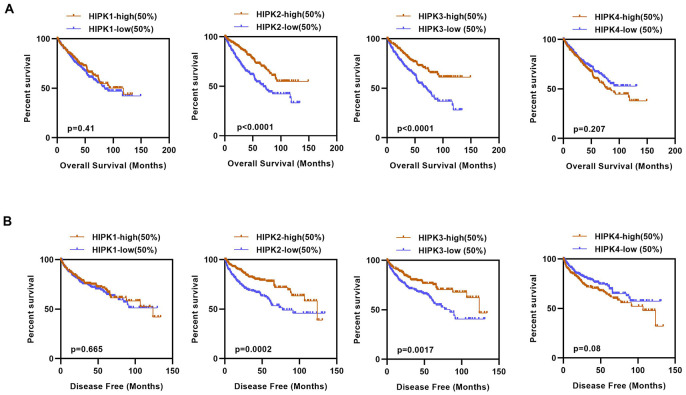
**Kaplan–Meier curves of OS and DFS in different expression levels of HIPK family.** (**A**) Lower HIPK2 and HIPK3 expressers had shorter OS than the higher expressers, HIPK1 and HIPK4 expression had no difference in OS. (**B**) Lower HIPK2 and HIPK3 expressers had shorter DFS than the higher expressers, HIPK1 and HIPK4 expression had no difference in DFS. OS, overall survival; DFS, disease–free survival; HIPK, Homeodomain interacting protein kinases.

**Figure 3:**
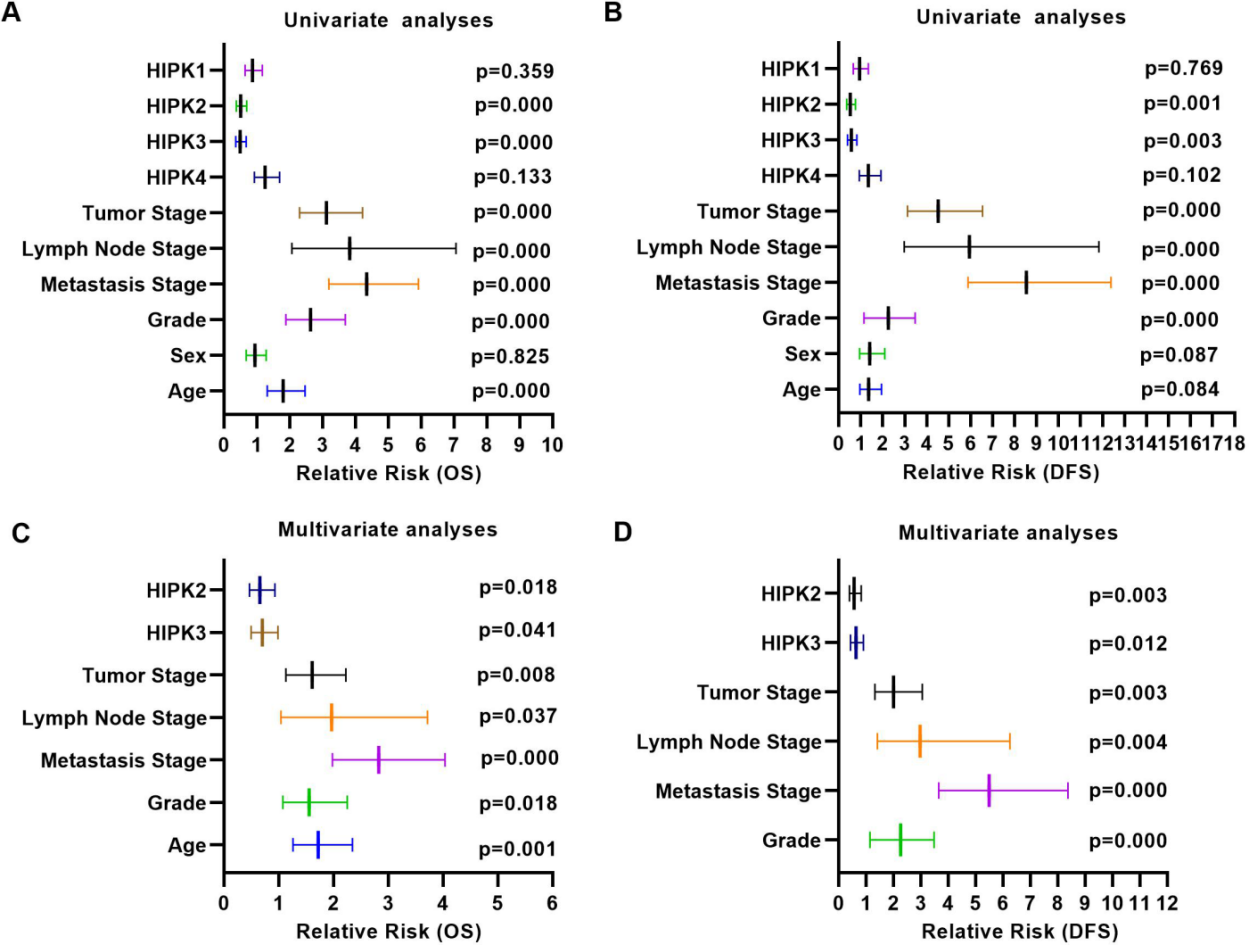
**Univariate analysis and multivariate analysis results of ccRCC with HIPKs expression.** (**A**) Univariate analysis showed that HIPK2 and HIPK3 were related to OS and DFS. (**A**) Multivariate analysis showed that HIPK2 and HIPK3 were related to OS and DFS. HIPK, Homeodomain interacting protein kinases.

We next investigated the prognostic value of HIPKs in ccRCC. As 525 patients had complete clinical data, patients were divided into high and low expression groups based on the median expression level of each HIPK member. Kaplan–Meier analysis shows shorter OS and DFS in patients with low HIPK2 or HIPK3 expression (Figure 2A, 2B). Univariate analysis showed that the expression of HIPK2 or HIPK3 was related to OS and DFS in Table 1. Then we selected the expression levels (high and low) of HIPKs, gender, age, N-stage, M-stage, T-stage, and grade for multivariate analysis of OS and DFS in Tables 2, 3. Univariate analysis and multivariate analysis results showed the prognostic indicators of HIPKs with OS and DFS in Figure 3A–3D. The multivariate analysis results are as follows: OS, age (HR, 1.722; P=0.001), T stage (HR, 1.613; P=0.008), N stage (HR, 1.966; P=0.037), M stage (HR, 2.829; P=0.000), Grade (HR, 1.558; P=0.018), HIPK2 (HR, 0.658; P=0.018) and HIPK3 (HR, 0.701; P=0.041). Meanwhile, multivariate analysis results showed the prognostic indicators of DFS: T stage (HR, 2.010; P=0.001), N stage (HR, 2.979; P=0.004), M stage (HR, 5.496; P=0.000), Grade (HR, 2.264; P=0.000), HIPK2 (HR, 0.567; P=0.003) and HIPK3 (HR, 0.630; P=0.012) could be considered as an independent prognostic indicators of DFS. Based on the above results, in the HIPK family, we identified that HIPK3 has a low mRNA expression in renal cancer tissues, and univariate and multivariate analysis showed that it can be used as a diagnostic marker. Then, we explored the possible role of HIPK3 in ccRCC.

**Table 1:** Comparison of OS and DFS between different expression levels of HIPK family.

**Variables**	**OS**		**DFS**	
	**χ^2^**	**P value**	**χ^2^**	**P value**
HIPK1 (high vs. low)	0.843	0.359	0.086	0.769
HIPK 2 (high vs. low)	18.903	0.000	11.827	0.001
HIPK 3 (high vs. low)	20.225	0.002	8.878	0.003
HIPK4 (high vs. low)	2.27	0.132	2.695	0.101

OS, overall survival; DFS, disease–free survival.

**Table 2:** Univariate and multivariate analyses of HIPK family mRNA level and patient overall survival.

**Variables**	**Univariate analysis**			**Multivariate analysis^c^**		
	**HR^a^**	**95%CI^b^**	**P value**	**HR^a^**	**95%CI^b^**	**P value**
Overall survival						
Age (years) ≤60 vs. >60	1.803	1.318-2.468	0.000	1.722	1.263-2.346	0.001
Sex Female vs. Male	0.948	0.697-1.290	0.825			
T stage T3 or T4 vs. T1 or T2	3.120	2.306-4.220	0.000	1.613	1.132-2.229	0.008
N stage N1 vs. N0 or NX	3.832	2.070-7.061	0.000	1.966	1.040-3.718	0.037
M stage M1 vs. M0 or MX	4.346	3.192-5.918	0.000	2.829	1.982-4.038	0.000
Grade G3 or G4 vs. G1 or G2	2.639	1.885-3.697	0.000	1.558	1.077-2.252	0.018
HIPK 1 High vs. Low	0.870	0.645-1.172	0.359			
HIPK 2 High vs. Low	0.513	0.377-0.697	0.000	0.658	0.466-0.930	0.018
HIPK 3 High vs. Low	0.499	0.366-0.680	0.000	0.701	0.499-0.985	0.041
HIPK 4 High vs. Low	1.258	0.932-1.699	0.133			

^a^Hazard ratio, estimated from Cox proportional hazard regression model.

^b^Confidence interval of the estimated HR.

^c^Multivariate models were adjusted for T, N, M classification, age and gender.

**Table 3:** Univariate and multivariate analyses of HIPK family mRNA level and patient disease–free survival.

**Variables**	**Univariate analysis**			**Multivariate analysis^c^**		
	**HR^a^**	**95%CI^b^**	**P value**	**HR^a^**	**95%CI^b^**	**P value**
Overall survival						
Age (years) ≤60 vs. >60	1.366	0.959-1.945	0.084			
Sex Female vs. Male	1.413	0.951-2.100	0.087			
T stage T3 or T4 vs. T1 or T2	4.526	3.134-6.538	0.000	2.010	1.322-3.057	0.001
N stage N1 vs. N0 or NX	5.942	2.983-11.836	0.000	2.979	1.419-6.254	0.004
M stage M1 vs. M0 or MX	8.529	5.877-12.379	0.000	5.496	3.605-8.378	0.000
Grade G3 or G4 vs. G1 or G2	3.376	2.236-5.098	0.000	2.264	1.147-3.485	0.000
HIPK 1 High vs. Low	0.948	0.666-1.352	0.769			
HIPK 2 High vs. Low	0.535	0.373-0.768	0.001	0.567	0.391-0.823	0.003
HIPK 3 High vs. Low	0.583	0.408-0.833	0.003	0.630	0.439-0.904	0.012
HIPK 4 High vs. Low	1.346	0.943-1.919	0.102			

^a^Hazard ratio, estimated from Cox proportional hazard regression model.

^b^Confidence interval of the estimated HR.

^c^Multivariate models were adjusted for T, N, M classification, age and gender.

### Clinicopathological parameters and molecular characteristics of HIPK3 in ccRCC patients

**Figure 4:**
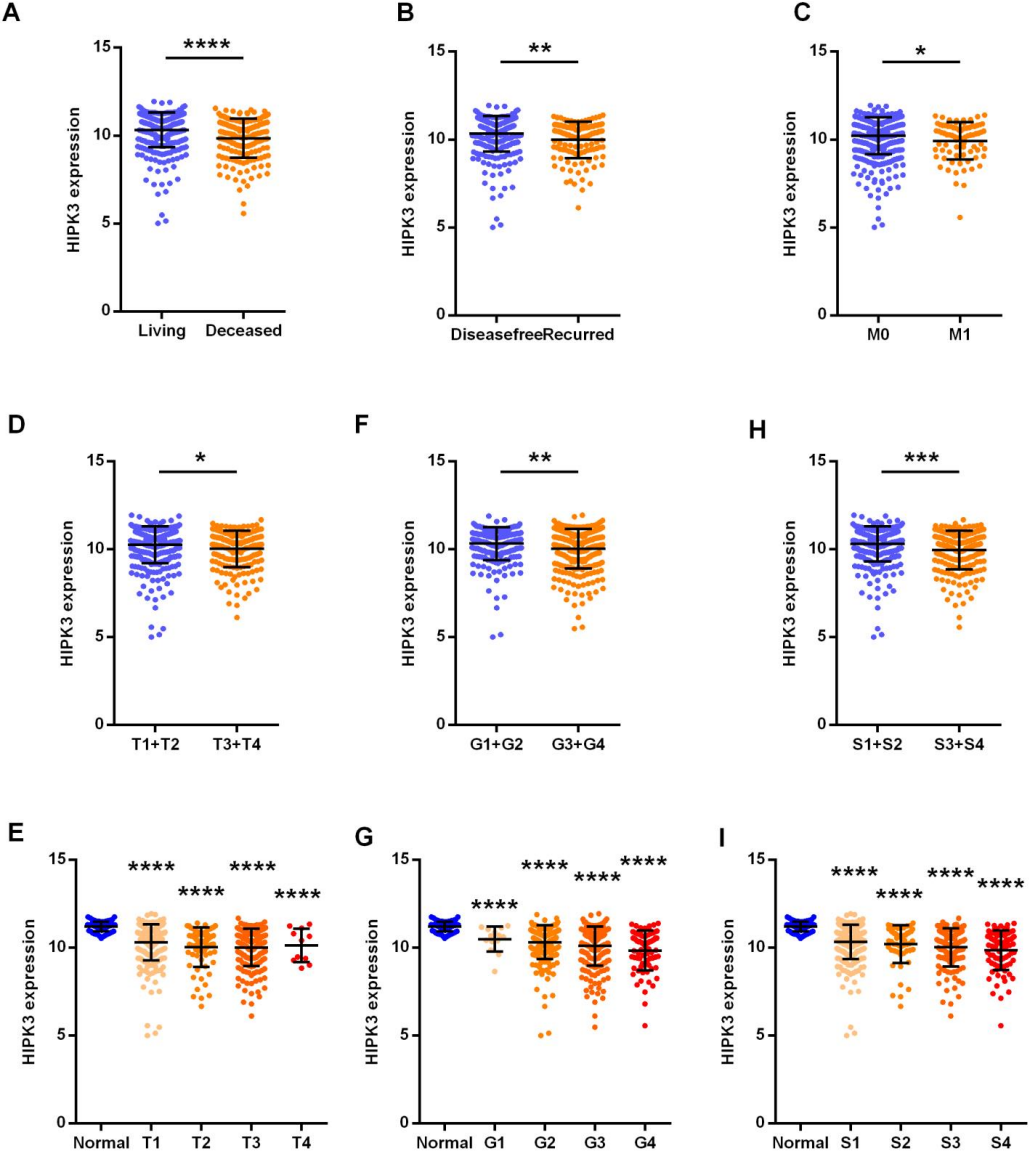
**HIPK3 correlated with various clinicopathological parameters in ccRCC tissues.** The mRNA levels of HIPK3 were compared in different clinicopathological parameters: (**A**) overall survival status, (**B**) disease free status cancer versus paired para-cancer, (**C**) M stage, (**D**) and (**E**) T stage, (**F**) and (**G**) grade, (**H**) and (**I**) TNM stage. S: TNM stage; HIPK, Homeodomain interacting protein kinases. *P<0.05, **P<0.01, ***P<0.001 and ****P<0.0001.

**Figure 5:**
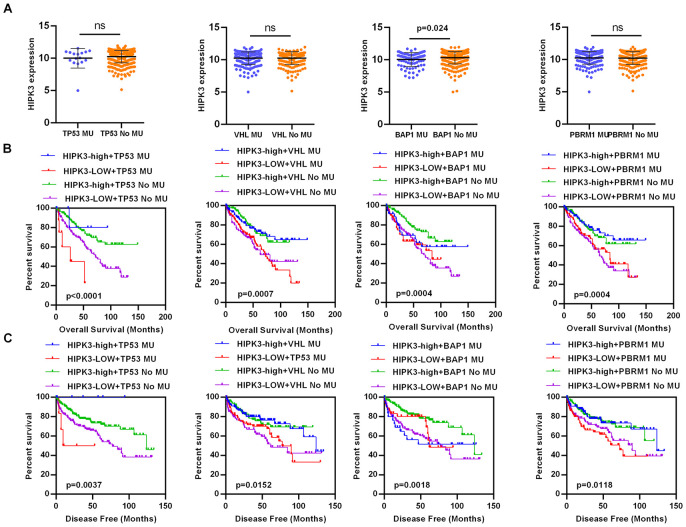
**HIPK3 expression is associated with BAP1 mutation.** (**A**) The expression of HIPK3 decreased when BAP1 mutation. (**B**, **C**) High expression of HIPK3 is correlate with good OS and DFS of renal cancer between wild-type and mutation subsets of TP53, VHL, BAP1, and PBRM1. OS, overall survival; DFS, disease–free survival; HIPK3, Homeodomain interacting protein kinases 3.

The clinical and pathological data of ccRCC patients are shown in Table 4. HIPK3 expression is related to tumor stage (T stage), distant metastasis (M stage), TNM stage and grade, but no significant differences in age, sex, or lymphatic metastasis (N stage). Significantly lower HIPK3 expression was found in dead patients (Figure 4A), recurred (Figure 4B), distant metastasis (Figure 4C), higher T stage (Figure 4D, 4E), higher grade (Figure 4F, 4G), higher TNM stages (Figure 4H, 4I) when compared to control patients. Analysis of these data indicates that HIPK3 expression is down-regulated in ccRCC and is significantly correlated with various clinicopathological parameters in ccRCC. TP53, VHL, BAP1, and PBRM1 have been showed to be common mutation in renal cancer. The expression of HIPK3 is downregulated with BAP1 mutation (Figure 5A). High expression of HIPK3 is correlate with good OS and DFS of renal cancer between wild-type and mutation subsets of these genes (Figure 5B, 5C).

**Table 4:** Correlation between HIPK3 mRNA expression and clinicopathological parameters of ccRCC patients.

**Variables**		**HIPK3 mRNA expression**			
		**Low (n=263)**	**High (n=262)**	**χ^2^**	**P value**
Age (years)	<=60	127	133		
	>60	136	129	0.321	0.601
Sex	male	85	162		
	female	178	100	1.967	0.171
T stage	T1+T2	190	147		
	T3+T4	72	116	15.783	0.000
N stage	N0+ NX	255	255		
	N1	8	7	0.065	1.000
M stage	M0+ MX	215	232		
	M1	48	30	4.798	0.037
Grade	G1+G2	108	137		
	G3+G4	155	125	6.645	0.011
TNM stage	I+II	141	178		
	III+IV	122	84	11.299	0.001

### The association between HIPK3 and diagnostic value in ccRCC

**Figure 6:**
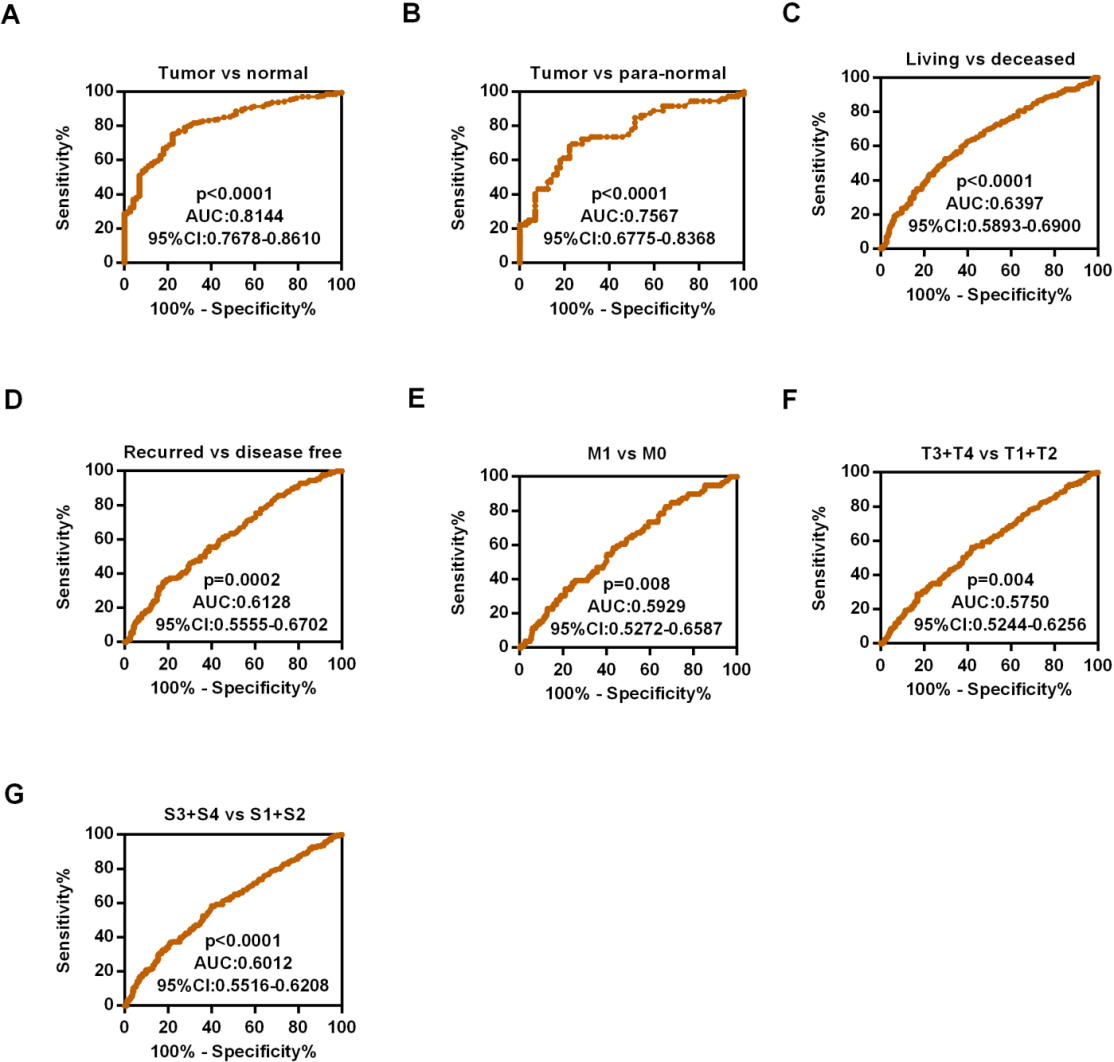
**Diagnostic efficiency of HIPK3 in ccRCC.** (**A**) ROC curve of HIPK3 between tumor and non-cancerous normal tissues, the AUC: HIPK3, 0.8144 (p<0.0001); (**B**) ROC curve of HIPK3 between tumor and paired non-cancerous normal tissues, the AUC: 0.7567, (p<0.0001); (**C**) Living vs deceased, AUC:0.6397, (p<0.0001); (**D**) Recurred vs disease free, AUC:0.6128, (p<0.0001). (**E**) M1 / M0 stage, AUC = 0.5929, (p=0.008). (**F**) (T3 + T4) / (T1 + T2), AUC = 0.5750, (p=0.004). (**G**) Pathological stage (III + IV)/ (I + II), AUC = 0.6012, (p < 0.0001). HIPK, Homeodomain interacting protein kinases; AUC, Area under the curve; ROC receiver operator characteristic.

As HIPK3 had a significantly different expression between tumor tissues and non-cancerous normal tissues, then we used ROC curves to analyze the diagnostic efficiency of HIPK3. The results showed that HIPK3 could distinguish ccRCC cancer from normal tissues statistically and produce the area under the curve (AUC) of 0.8144 (95% CI: 0.7678-0.8610; p < 0.0001, Figure 6A), ccRCC of para-normal tissue with AUC of 0.7567 (95% CI: 0.6775-0.8368; p < 0.0001, Figure 6B). In addition, a subgroup ROC curve analysis of clinical characteristics suggests that low expression of HIPK3 may have diagnostic value in ccRCC patients with living vs deceased (AUC = 0.6397, 95% CI: 0.5893-0.6900, p < 0.0001, Figure 6C), recurred vs disease free (AUC = 0.6128, 95% CI: 0.5555-0.6702, p=0.0002, Figure 6D), M1 / M0 stage (AUC = 0.5929, 95% CI: 0.5175-0.6587, p=0.008, Figure 6E), (T3 + T4) / (T1 + T2) (AUC = 0.5750, 95% CI: 0.5244-0.6256 p=0.004, Figure 6F), pathological stage (III + IV)/ (I + II) (AUC = 0.6012, 95% CI: 0.5516-0.6206, p < 0.0001, Figure 6G).

### Confirm of the expression levels of HIPK3 in RCC tissues

**Figure 7:**
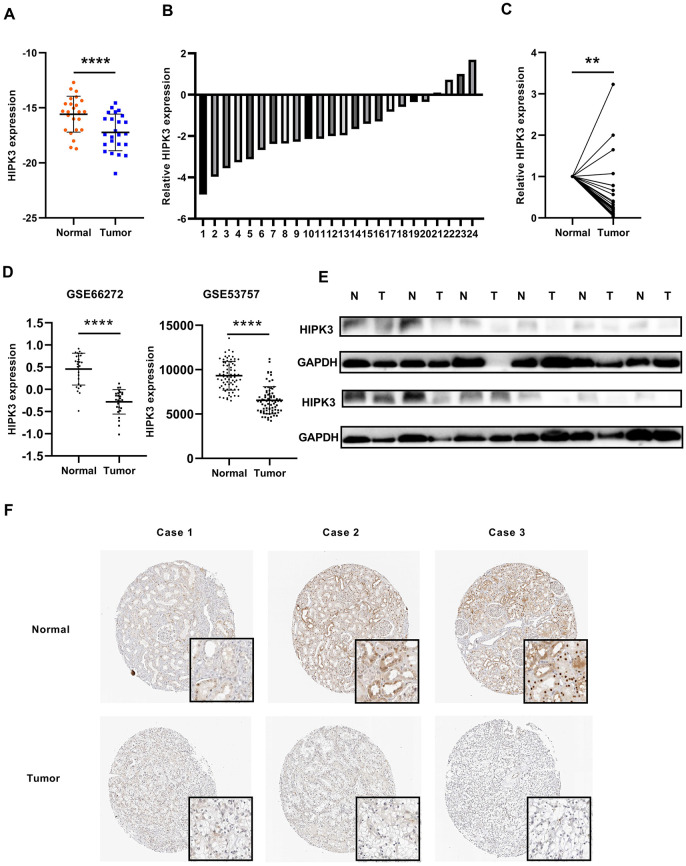
**HIPK3 is downregulated in ccRCC tissues.** (**A**, **B**) Gene expression levels of HIPK3 in renal cancer tissues. (**C**) Relative HIPK3 expression was downregulated in renal cancer tissues. (**D**) Validation of HIPK3 expression in renal cancer with Gene Expression Omnibus (GEO66727, GEO53757). (**E**) IHC analyses of HIPK3 expression in ccRCC tissues and paracancer tissues. **P<0.01 and ****P<0.0001.

We used quantitative real-time polymerase chain reaction (qRT-PCR) to validate the results of the database. The patient's clinical information is shown in Supplementary Table 1. The results showed that the mRNA expression of HIPK3 was significantly down-regulated in ccRCC in Figure 7A, 7B, the relative expression levels of HIPK3 were significantly lower in RCC tissues (Figure 7C). Further validation of HIPK3 with Gene Expression Omnibus (GEO66727, GEO53757) showed that the downregulation of HIPK3 in renal cancer (Figure 7D). Western blotting of 12 paired ccRCC tissues showed that the protein of HIPK3 was downregulated in renal cancer (Figure 7E). Immunohistochemistry (IHC) results also showed that HIPK3 was downregulated in renal cancer tissues. (Figure 7F). These results indicate that HIPK3 is down-regulated in RCC cancer tissues and this is consistent the results in TCGA database.

### Overexpression of HIPK3 can inhibit the proliferation, migration, and invasion of renal cancer cells in vitro

**Figure 8:**
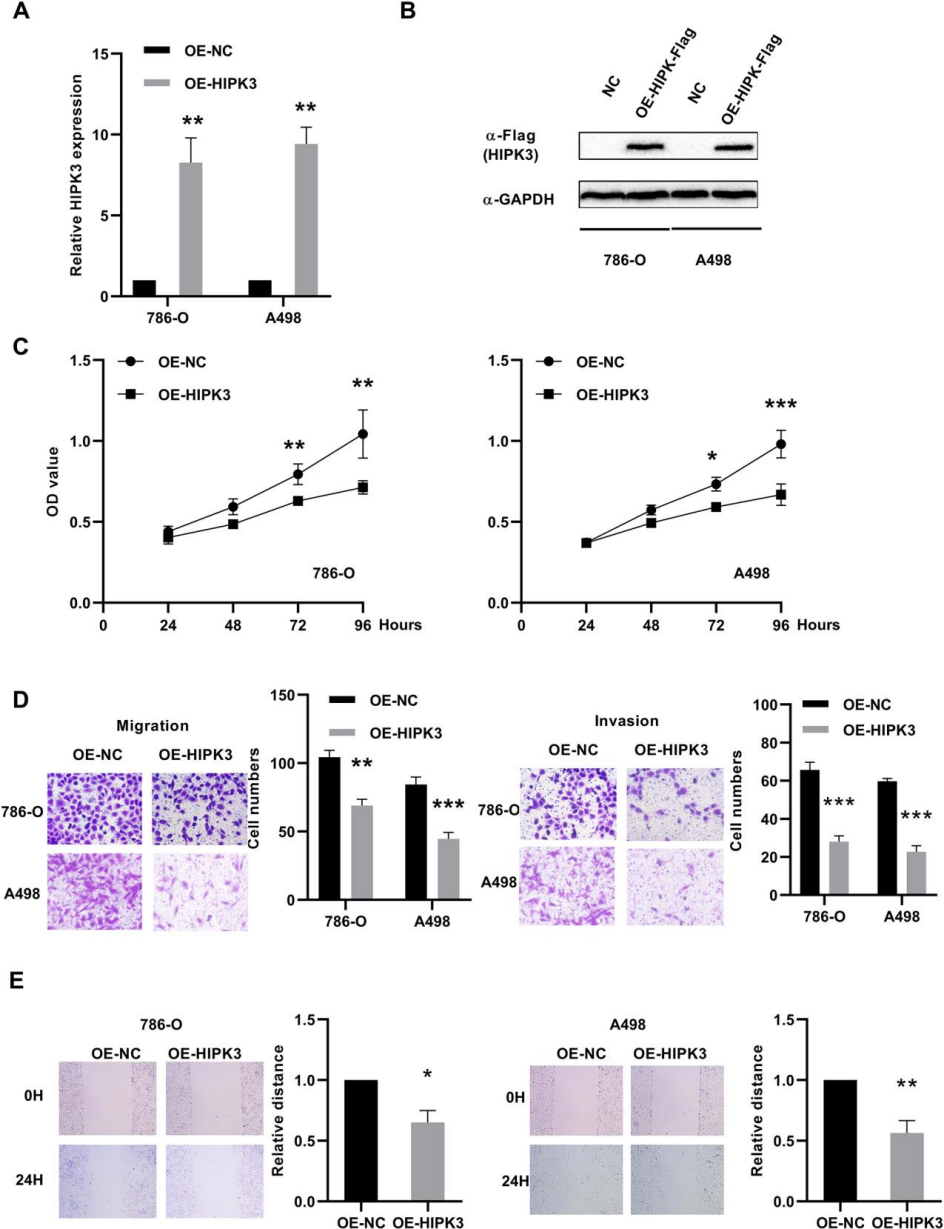
**Effects of HIPK3 overexpression on cell proliferation, migration, and invasion.** (**A**, **B**) HIPK3 mRNA and protein expressions were successfully overexpressed in 786-O and A498 cells. (**C**) Cell counting kit-8 assay detected the effects of HIPK3 overexpression on the proliferation of 786-O and A498 cells. (**D**, **E**) Representative images of migration and invasion assays performed using 786-O and A498 cells with transwell and wound healing assay. Data are presented as the mean ± SD from three independent experiments. *P<0.05, **P<0.01, and ***P<0.001.

To investigate the possible role of HIPK3 in renal cancer cells, we transfected HIPK3 overexpression (OE-HIPK3) and OE-NC plasmids into 786-O and A498 cells to test the effect of HIPK3 on cancer cell growth in vitro (Figure 8A, 8B). According to the analysis of CCK8 test results, HIPK3 overexpression can inhibit the proliferation function of renal cancer cells (Figure 8C). HIPK3 repressed the migration and invasion capability of 786-O and A498 cells with transwell and wound healing assay (Figure 8D, 8E). The inhibitory effect of HIPK3 on tumors may be related to the expression of twist1, cdh1 (E-cadherin), and vimentin.in Supplementary Figure 1.

## DISCUSSION

The HIPK family comprises 4 members, HIPK1-4, can regulate cell proliferation and apoptosis [17], but has not been study in ccRCC. In the current research, we explored role of HIPKs in kidney cancer. Among the 4 HIPKs, HIPK1 and HIPK3 mRNA expression was downregulated in cancer tissues from The Cancer Genome Atlas Kidney Clear Cell Carcinoma (TCGA-KIRC) database. Kaplan–Meier analysis confirmed that patients with low HIPK2 or HIPK3 expression had poor OS and DFS. Univariate analysis and multivariate analysis showed that the expression of HIPK2 or HIPK3 was associated with OS and DFS. Based on the above results, we believe that HIPK3 has an important role in the occurrence, development of ccRCC.

Because HIPKs can induce proliferation, but also can lead to cell death, its role in cancer cells may be multifaceted. The extensive research results of HIPKs and p53 have suggested that HIPKs can suppress tumors in response to ionizing radiation [18]. HIPK2 works synergistically with p53 to suppress ray-induced thymoma by facilitating tumor cell death [19]. HIPKs expression has been found to be reduced in a variety of cancers, such as breast cancer [20], idiopathic pulmonary fibrosis [21], thyroid cancer [22].

Guang et al. found that HIPK2 can inhibit epidermal stem cell expansion and skin tumors [23]. Nodale et al. reported that HIPK2 can suppress breast cancer by inhibiting vimentin [24]. D’ Orazi et al. found that HIPK2 inhibits the development of human colon tumors [25]. In our study, HIPK2 did not have a significant difference in renal cancer tissues, but its low expression predicted a poor prognosis, and the univariate analysis and multivariate analysis also indicates that low expression may be an independent prognostic marker for ccRCC.

Curtin et al. investigated the possibility of HIPK3 and FAS-mediated apoptosis in prostate cancer [26]. Liu et al. confirmed that HIPK3 in NSCLC tissues was downregulated, and low HIPK3 expression significantly correlated with poor survival. It has been reported that HIPK3 can be a valuable biomarker for the survival prognosis of patients with non-small cell lung cancer [20]. Our research results showed HIPK3 had a significantly lower expression in renal cancer tissues, and its low expression predicted a poor prognosis, and univariate analysis and multivariate analysis also indicates that low expression may be a novel independent prognostic marker for ccRCC.

In this study, we first analyzed the relationship between HIPK family expression and the clinicopathological characteristics of tumors and patient survival in TCGA-KIRC. HIPK3 expression level is closely related to T-stage, M-stage, TNM-stage, and grade. These results suggest that HIPK3 expression level may be an independent predictor of OS and DFS prognosis. ROC curve analysis found that the distinguish role in ccRCC from normal individuals with expression of HIPK3. Then we verified the results of the database through experiments on samples of clinical cases. *In vitro* experiments showed that overexpression of HIPK3 can inhibit tumor cell growth, invasion, and migration. VHL, PBRM1, BAP1, and TP53 have been showed to be common mutation in renal cancer [8]. The expression of HIPK3 decreased when BAP1 mutation. High expression of HIPK3 is correlate with good OS and DFS of renal cancer between wild-type and mutation subsets of TP53, VHL, BAP1, and PBRM1. Loss of HIPK1 and HIPK2 leads to upregulation of angiogenic genes [27]. Qin et al. showed that HIPK2 could suppress pancreatic cancer proliferation in part of inhibiting the ERK/cMyc axis [28]. ERK pathway was associated with poor prognosis of ccRCC patients, HIPK3 may have the anti-angiogenesis thought ERK pathway. HIPK1 acted as a novel kinase which was required for optimal B cell function [29]. HIPK2 deficiency impaired IFN production in macrophages [30]. HIPK was closely related to immunity. However, the detailed mechanism of action of HIPK3 and these molecules has not been reported, which is worthy of future study.

## CONCLUSIONS

Here we investigated the prognostic role of HIPK family in TCGA-KIRC and found that HIPK3 is an independent predictor of prognosis in ccRCC. HIPK3 expression was lower in TCGA-KIRC database and our own clinical cases. Cell experiments showed that HIPK3 could inhibit the malignant characteristics of tumors. The above results indicate that HIPK3 may be a potential new biomarker for predicting the prognosis of patients with ccRCC and become a new therapeutic target for ccRCC.

## MATERIALS AND METHODS

### Patient samples

A total of 533 patients were included in the Cancer Genome Atlas Renal Clear Cell Carcinoma (TCGA-KIRC) database, of which 525 patients had complete information for univariate and multivariate Cox proportional hazard regression [31]. Immunohistochemistry assays of renal cancer patients were download from the Human Protein Atlas (https://www.proteinatlas.org/). Further validation of HIPK3 was obtained from Gene Expression Omnibus (GEO66727, GEO53757) database (http://www.ncbi.nlm.nih.gov/geo/). Twenty-four pairs of surgical specimens from ccRCC patients were collected from Department of Urology, Union Hospital, Tongji Medical College, Huazhong University of Science and Technology from 2016 to 2019. All patients have been notified and obtained informed consent, and all trial content and procedures have been approved by the Institutional Review Board of Huazhong University of Science and Technology for experimental and research procedures.

### RNA extraction and qRT-PCR

RNA extraction of tissue and cell was used with TRizol reagent (Thermo, Massachusetts, USA) and qPCR analysis was performed as previous study [32]. HIPK3 expression was calculated by: 2^-ΔCt^ (ΔCt = Ct_HIPK3_–Ct_GAPDH_).

GAPDH (forward, 5′-GAGTCAACGGATTTGGTCGT-3′;

reverse, 5’-GACAAGCTTCCCGTTCTCAG-3′)

HIPK3, (forward, 5′-ACATTGGAAGAGCATGAGGCAGAGA-3′,

reverse, 5′-CTGCTGAAAAGCATCACCACAACCA-3′)

### Cell culture

786-O and A498 were obtained from the American Type Culture Collection and cultured in high glucose DMEM medium (Wuhan Boster Biological Technology, Ltd., Wuhan, China) which contained 10% FBS (Gibco; Thermo Fisher Scientific, Inc., Waltham, MA, USA).

### Cell proliferation, migratory and invasion assays

Plasmid of overexpression of HIPK3 (OE-HIPK3,) or negative control (OE-NC) were obtained from GeneChem. Transfected the plasmid into 786-O and A498 cells with Lipofectamine 3000, and then the cells were added 3x10^3^/well to the 96-well plate. Cell Counting Kit-8 (CCK-8) (Dojindo Molecular Technologies, Inc, Rockville, MD, USA) was used to determine cell proliferation rate (OD value). For migratory and invasion assay, 10^5^ cells or 2 x 10^5^ were seeded in transwell plate with polycarbonate membrane inserts without or with Matrigel (Corning, New York, USA). After 24 hours, cells were fixed, stained, and counted as previous study. For wound healing assay, cells were grown in six-well plates and wounded by a 10 μl pipette tip. Then, remove cell debris and images were taken at time 0/ 24 h [33].

### Western blotting

Proteins of renal cancer tissues and cells are pyrolyzed, separated and transferred to membrane, blocked and detected as previously described with GAPDH (Wuhan Boster Biological Technology, 1:2000; BM3876), HPIK3 (Proteintech, 1:1000) and Flag (Abclonal, AE005, 1:1000) [34].

### Statistical analysis

The RNA results of the paired samples in the data were analyzed by the paired samples t-test, and the unpaired and all overall samples were analyzed by the t-test. The area under the curve (AUC) and receiver operator characteristic (ROC) curves were used to analyze clinical differential diagnosis results and diagnostic efficiency. The Kaplan-Meier (KM) curve evaluated the relationship between the clinical survival rate and the expression level of HIPKs with log-rank test. The prognostic significance of HIPKs was analyzed by univariate and multivariate Cox proportional hazards regression in ccRCC as previously described [35]. P <0.05 was considered statistically significant. All statistical analysis results are performed by SPSS Statistics 22.0 (IBM SPSS, Chicago, IL).

## Supplementary Material

Supplementary Figure 1

Supplementary Table 1
