# Supplementary material for: Identification of HIPK3 as a potential biomarker and an inhibitor of clear cell renal cell carcinoma
Source: Aging (Albany NY). 2021 Jan 20;13(3):3536–53. doi: 10.18632/aging.202294 (PMC7906163; doi:10.18632/aging.202294)
Supplement: Supplementary Figure 1 [file aging-13-202294-s001.pdf]

## SUPPLEMENTARY FIGURE

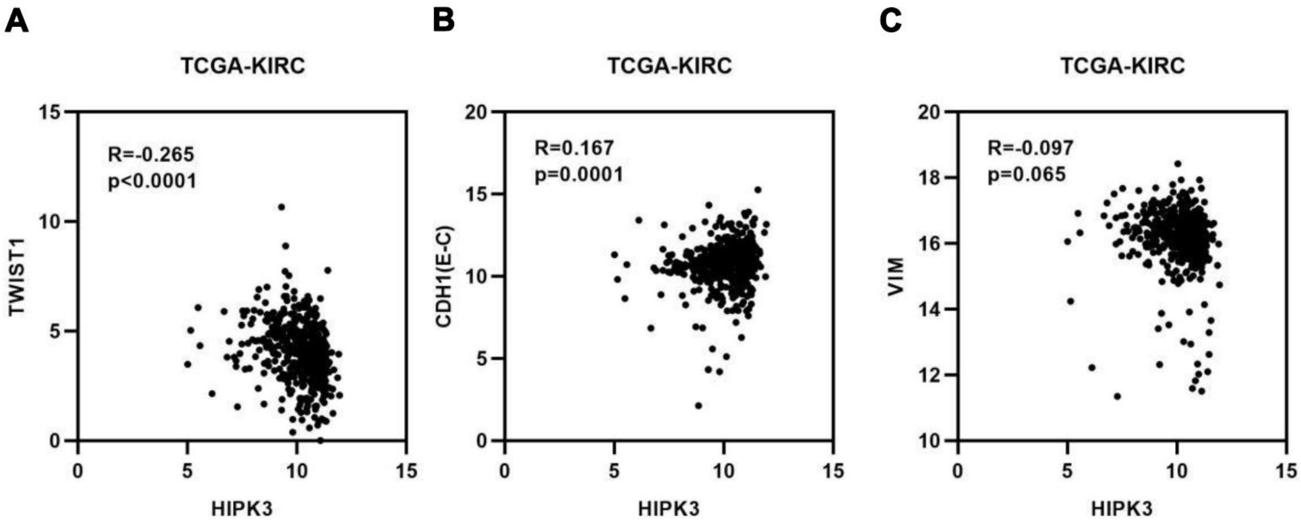


**Supplementary Figure 1. Correlation analysis of HIPK3 and tumor-related indicators in the Cancer Genome Atlas.** (**A**) Twist1, (**B**) CDH1(E-Cadherin), (**C**) Vimentin.
