# Supplementary material for: Identification of HIPK3 as a potential biomarker and an inhibitor of clear cell renal cell carcinoma
Source: Aging (Albany NY). 2021 Jan 20;13(3):3536–53. doi: 10.18632/aging.202294 (PMC7906163; doi:10.18632/aging.202294)
Supplement: Supplementary Table 1 [file aging-13-202294-s002.pdf]

SUPPLEMENTARY TABLE

Supplementary Table 1. Clinicopathological parameters of 24 ccRCC patients.

Variables	validation set<br>24 pairs of ccRCC and adjacent normal tissues
Gender (no.) male, female,	24
Pathological T stage no.	
T1	6
T2	11
T3	7
T4	0
Pathological N stage no.	
N0	22
N1	2
Pathological M stage no.	
M0	22
M1	2
Pathological grade no.	
G1	7
G2	12
G3	5
G4	0
AJCC TNM stage no.	
I	8
II	10
III	6
VI	0
